# Targeted Glutamate Supply Boosts Insulin Concentrations, Ovarian Activity, and Ovulation Rate in Yearling Goats during the Anestrous Season

**DOI:** 10.3390/biology12071041

**Published:** 2023-07-24

**Authors:** Luis A. Luna-Garcia, Cesar A. Meza-Herrera, Carlos C. Perez-Marin, Angeles De Santiago-Miramontes, Jessica M. Flores-Salas, Rebeca Corona, Guadalupe Calderon-Leyva, Francisco G. Veliz-Deras, Cayetano Navarrete-Molina, Ruben I. Marin-Tinoco

**Affiliations:** 1 Unidad Regional Universitaria de Zonas Aridas, Universidad Autonoma Chapingo, Bermejillo, Durango 35230, Mexico; 2 Department of Animal Medicine and Surgery, Faculty of Veterinary Medicine, University of Cordoba, 14014 Cordoba, Spain; 3 Programa de Posgrado en Ciencias en Produccioon Agropecuaria, Universidad Autonoma Agraria Antonio Narro, Periferico Raúl López Sanchez y Carretera a Santa Fe, Torreon 27054, Mexico; 4 Departamento de Neurobiologia Celular y Molecular, Laboratorio de Neuroanatomia Funcional y Neuroendocrinologia, Instituto de Neurobiologia, UNAM, Queretaro 76230, Mexico; 5 Department of Chemical and Environmental Technology, Technological University of Rodeo, Durango 35760, Mexico

**Keywords:** goats, glutamate, insulin, ovarian activity, anestrous

## Abstract

**Simple Summary:**

Our research question considered whether exogenous glutamate administration would enhance serum insulin levels linked to augmented ovarian activity in yearling goats during the anestrous season. The obtained research outcomes support our initial working hypothesis. Certainly, we found that glutamate evokes serum insulin increases across time, enhancing in parallel the out-of-season ovarian activity, which is an augmented ovulation rate in yearling goats.

**Abstract:**

The neuroendocrine regulation of the seasonal reproductive axis requires the integration of internal and external signals to ensure synchronized physiological and behavioral responses. Seasonal reproductive changes contribute to intermittent production, which poses challenges for optimizing goat product yields. Consequently, a significant objective in seasonal reproduction research is to attain continuous reproduction and enhance profitability in goat farming. Glutamate plays a crucial role as a modulator in several reproductive and metabolic processes. Hence, the aim of this study was to evaluate the potential impact of exogenous glutamate administration on serum insulin concentration and ovarian function during the out-of-season period in yearling goats. During the anestrous season, animals were randomly located in individual pens to form two experimental groups: (1) glutamate (n = 10, live weight (LW) = 29.1 ± 1.02 kg, body condition score (BCS) = 3.4 ± 0.2 units) and (2) control (n = 10; LW = 29.2 ± 1.07 kg, BCS = 3.5 ± 0.2), with no differences (*p* < 0.05) regarding LW and BCS. Then, goats were estrus-synchronized, and blood sampling was carried out for insulin quantification. Ovaries were ultrasonographically scanned to assess ovulation rate (OR), number of antral follicles (AFs), and total ovarian activity (TOA = OR + AF). The research outcomes support our working hypothesis. Certainly, our study confirms that those yearling goats treated with exogenous glutamate displayed the largest (*p* < 0.05) insulin concentrations across time as well as an augmented (*p* < 0.05) out-of-season ovarian activity.

## 1. Introduction

Changes in biological rhythms play a crucial role as adaptive and evolutionary mechanisms that promote reproductive success, individual survival, and species perpetuation [1]. Seasonal breeders show modifications in their physiology and behavioral responses to effectively adapt to seasonal variations in the environment. Among the various environmental cues, photoperiod stands out as the most reliable indicator for temporal prediction throughout the year [2]. This intricate process occurs through the retino-hypothalamic tract, a complex polysynaptic pathway that transforms external signals into neurohormonal signals. These neurohormonal signals subsequently act on specific brain nuclei responsible for the regulation of biological rhythms, resulting in increased melatonin release [3]. Melatonin levels affect reproductive function by regulating both the frequency and measure of reproductive hormones, which are regulated by the expression of melatonin receptors in hypothalamic nuclei that are involved in the control of GnRH release [1,2]. GnRH is the key master hormone in the hypothalamic–pituitary–gonadal (HPG) axis. GnRH neurons in the preoptic area release GnRH near the pituitary portal capillaries at the median eminence in a pulsatile manner modulating the HPG axis, which subsequently stimulates the pulsatile release of gonadotropins, i.e., luteinizing hormone (LH) and follicle-stimulating hormone (FSH) from the anterior pituitary [4,5].

Gonadotropins drive gametogenesis in response to the synthesis of sex steroids and through feedback actions modulate the activity of GnRH in the HPG axis [6]. Ovarian steroids influence the reproductive system, but it is susceptible to other signals such as photoperiod, breed, age, stress, and metabolic status [7]. GnRH can be modulated in several ways by different molecules (i.e., neuropeptides and neurotransmitters); among these, kisspeptin is the essential molecule in the control of reproductive function, integrating hormonal, nutritional, and metabolic cues [5,8,9]. Kisspeptin can interact with other neuropeptides, hormones, and neurotransmitters to achieve its actions, including excitatory and inhibitory amino acids, such as glutamate and GABA, respectively [10]. 

Glutamate is the main excitatory neurotransmitter in the central nervous system (CNS) and is involved in a myriad of brain functions, acting through kindred receptors expressed in central brain areas, including those related to reproductive function [11,12]. Numerous studies investigating the effect of glutamate have demonstrated its ability to modulate the HPG axis. Glutamate, along with its agonist, has been shown to increase the release of key reproductive hormones, including GnRH and LH [13,14]. Additionally, glutamate has been observed to act on non-neuronal cells, stimulating the release of gliotransmitters that in turn impact GnRH release [15]. Both glutamate and its receptors have been reported outside the CNS in various tissues such as the liver, kidney, skeletal muscle, and pancreatic islets, participating in diverse metabolic pathways [11,12]. The importance of exogenous glutamate has been demonstrated in previous works of our research team; it modulates reproductive neuroendocrine functions in small ruminants, such as puberty, sexual behavior, and reactivation of the reproductive axis and the out-of-season ovarian function, being an alternative for reproductive management [16,17,18,19]. 

Currently, achieving enhanced animal production and reproduction while simultaneously considering animal welfare and sustainable production has become a significant challenge. One key aspect of this challenge involves reducing the reliance on exogenous hormones in these processes [20,21,22]. Building on such findings, we hypothesized that the administration of exogenous glutamate promotes increased serum insulin levels and, in turn, promotes an augmented ovarian function in yearling goats during the anestrous season. Therefore, this study was designed to validate such a working hypothesis.

## 2. Materials and Methods

The research conducted in this study adhered strictly to established guidelines for the ethical treatment, care, and welfare of animals in research at both national [23] and international [24] levels. All procedures, methods, and management of the animals were carried out following these recognized recommendations. This study received institutional approval under the reference number UACH-DGIP-REBIZA-IBIODEZA/15-510-400-2.

### 2.1. Study Location, Environmental Conditions, Animal Selection, and Management

The research was conducted in the northern region of Mexico (26° N, 103° W; 1120 m). This study involved 20 yearling Alpine-Saanen-Nubian x Criollo goats in anestrus condition. The goats had an average live weight (LW) of 29.17 ± 1.02 kg and a body condition score (BCS) of 3.45 ± 1.02 units. This study was conducted during April, May, and June, which corresponded to the natural deep anestrous at 26° N. Before feeding, both the LW and BCS were recorded weekly. The body condition score was determined by an experienced technician using a five-point scale ranging from 1 (emaciated) to 5 (obese).

### 2.2. Experimental Design and Treatments

**Figure 1:**
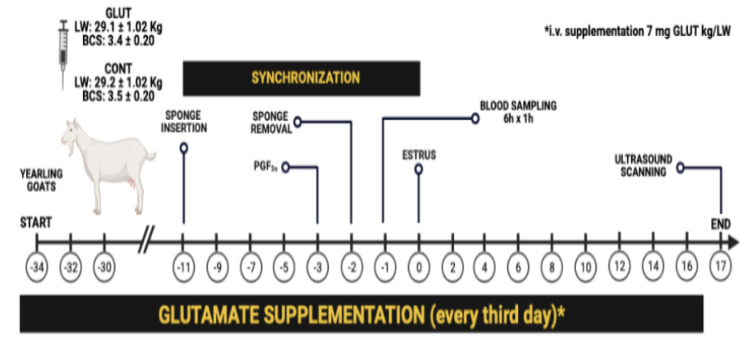
Timeline of activities performed during the experiment. Glutamate was supplemented (i.v. L-glutamate) every three days from d34 pre-estrus to d17 post-estrus throughout the experimental period. While the estrus synchronization protocol was conducted, an intensive blood sampling for insulin quantification was performed at 1 h intervals for 6 h, one day before the estrus. Ovarian ultrasonographic scanning was carried out on d17 post-estrus to assess the relationship between the TOA and serum insulin concentration. All the experimental groups had *ad libitum* water access and shaded areas in each pen.

At the beginning of May, the animals were randomly assigned to individual pens forming two distinct experimental groups: (1) glutamate (GLUT; n = 10, LW = 29.1 ± 1.02 kg, BCS = 3.4 ± 0.2 units) and (2) control (CONT; n = 10; LW = 29.2 ± 1.07 kg, BCS = 3.5 ± 0.2 units). Both groups had similar average LW and BCS. The GLUT goats received an intravenous dose of 7 mg/kg of glutamate (L-glutamate, Merck-C5H9NO4-art-101791, Darmstadt, Germany) every third day throughout the entire experimental period, which lasted from 34 d pre- to 17 d post-estrus. The experimental groups were provided a basal diet twice daily (at 07:00 and 16:00 h). The diet consisted of a mixture of alfalfa hay (14% crude protein (CP), 4.7 net energy for maintenance (NEm) MJ kg^−1^), corn silage (8.1% CP, 6.7 NEm MJ kg^−1^), and corn grain (11.2% CP, 9.9 NEm MJ kg^−1^), balanced to meet their net energy requirements for maintenance [25]. Both groups had *ad libitum* access to water and shaded areas. The composition values of the components in the basal diet (dry matter; % basis) were determined. Random samples of the diet were collected and subjected to further processing. The samples were initially reduced by scratching and finely chopped on clean paper. Subsequently, the processed samples were sieved using a 1 mm mesh sieve [26]. The specific activities conducted during the experimental period are illustrated in Figure 1.

### 2.3. Estrus Synchronization, Blood Sampling, and Insulin Measurement

On day −11 (according to Figure 1), i.e., 23 days after the beginning of this experiment, the estrus synchronization treatment was initiated using intravaginal sponges containing 45 mg of fluorogestone acetate (Chronogest^®^; Intervet International B.V., Boxmeer, Holland). The sponges were left in place for 10 days. Nine days after sponge insertion (day −3; day 0 = estrus), goats received a single intramuscular dose of 1 mL prostaglandin F2α (0.075 mg D-cloprostenol/goat; Prosolvin-C^®^, Intervet International B.V., Boxmeer, Holland). Subsequently, on day −2, the sponges were removed. Twenty-four hours later (day −1), five goats from each group were randomly selected for intensive blood sampling. Blood samples were collected, by jugular vein puncture, using sterile tubes (Corvac; Kendall Health Care, St. Louis, MO, USA) every hour for 6 h, starting 3 h after the morning feeding. Serum was separated by centrifugation (1500× *g*, 15 min), decanted, transferred to polypropylene microtubes (Axygen Scientific, Union City, CA, USA), and stored at −20 °C until hormone analysis. Serum insulin concentrations were determined in duplicate by radioimmunoassay (RIA) in blood serum using components of a commercial solid-phase RIA kit (Diagnostic Products, Los Angeles, CA, USA), as previously outlined [27]. Basal insulin levels were quantified as the mean of the lowest points observed during the defined sampling period [28,29]. The intra-assay coefficient of variation value for insulin quantification was 10.0% with a detection limit of 0.2 ng mL^−1^.

### 2.4. Ultrasonographic Scanning of Ovarian Activity

Transrectal ultrasonography was performed on all the goats included in the GLUT group (n = 10) and CONT group (n = 10) using a 7.5 MHz linear array transducer (Toshiba Medical Systems Ltd., Crawley, UK). A qualified operator performed the ultrasound scans on day 17 post-estrus, coincident with the end of luteal phase in mid-May, to assess ovarian activity. Each ovary was meticulously examined and the number of corpora lutea (CL) and antral follicles (AFs) were recorded following established procedures [30]. Subsequently, total ovarian activity (TOA) was calculated by summing the recorded numbers of AF and CL from both ovaries in each animal in the experimental group. Together, this composite measurement provided an overall assessment of ovarian activity throughout the study.

### 2.5. Statistical Analyses

A split-plot ANOVA for repeated measures was used to evaluate the effects of live weight, body condition score, ovulation rate, total ovarian activity, and serum insulin concentrations over time within the same animal. The main plot included treatments, with within-treatment animals as the error term. The subplot consisted of time and time × treatment, which were tested using the residual mean square [31]. Where significant F-values were observed, mean separation was performed using the LSMEANS-PDIFF option of PROC GLM. To assess normality, response variables were subjected to the Shapiro–Wilk test. Log10 transformation was applied to baseline and mean serum insulin concentrations to address the skewness of the data. When a significant interaction between treatment and time was detected, comparisons were made between different time points. These statistical analyses were performed using SAS GLM procedures (SAS Inst. Inc., V9.1, Cary, NC, USA). Pearson correlations were used to examine associations between live weight, body condition score, and number of luteal structures. Non-transformed data are presented for ease of interpretation and are expressed as least squares means ± standard error (SE). The most conservative SE is indicated, and the threshold for statistical significance was set at 0.05.

## 3. Results

No differences between experimental groups (*p* > 0.05) occurred when comparing LW and BCS, neither at the beginning (29.4 ± 1.02 kg and 3.4 ± 0.17) nor at the end of the experimental period (i.e., 35.13 ± 1.07 kg and 3.4 ± 0.2 units). Significantly greater OR (1.77 ± 0.20; *p* < 0.05) and TOA (4.11 ± 0.47; *p* < 0.05) were present in the GLUT-treated animals compared to the control animals (Table 1).

**Figure 2:**
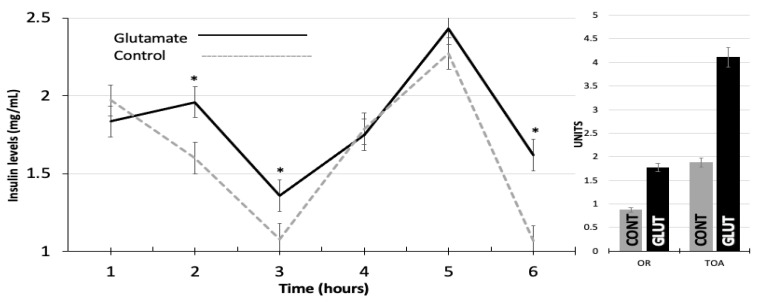
Serum insulin concentrations (mg mL^−1^) across time (**left panel**) and ovulation rate (units) and total ovarian activity (units) (**right panel**) in glutamate-supplemented (GLUT) and non-supplemented (CONT) goats during the non-breeding season. * Indicates differences (*p* < 0.05) between treatments across time.

Concerning the response variable serum insulin concentrations across time, the phenotypic values also favored (*p* < 0.05) the GLUT-treated group. Both the serum insulin concentrations across the experimental period along with the OR and TOA are shown in Figure 2. Positive correlations occurred between LW and BCS (r^2^ = 0.71; *p* < 0.01), BCS and TOA (r^2^ = 0.7, *p* < 0.05), and AF and OR (r^2^ = 0.61, *p* = 0.01), as well as between OR and TOA (r^2^ = 0.87, *p* = 0.001).

## 4. Discussion

Our research question considered whether exogenous glutamate administration would enhance serum insulin levels linked to augmented ovarian activity in yearling goats during the anestrous season. Certainly, the outcomes support the initial working hypothesis, and we observed that glutamate evokes serum insulin increases over time, enhancing in parallel the out-of-season ovarian activity in yearling goats. It is noteworthy that the differences observed in serum insulin levels among the groups were not influenced by LW and BCS, as there were no variations between the experimental groups for these variables. Based on our research findings, the increase in both serum insulin levels and ovarian activity in glutamate-treated goats indicates that the administration of exogenous glutamate to yearling goats during seasonal anestrous diminishes the inhibitory feedback of estradiol at the hypothalamic level. This, in turn, led to an increase in ovarian activity. This neuroendocrine response suggests an activation of the hypothalamic non-neuronal cells. On the other hand, exogenous glutamate administration suggests an augmented release pattern of the pancreatic release of insulin, accompanied by increases in the ovarian function of the glutamate-supplemented group. A third possible scenario involves a direct effect of exogenous glutamate on ovarian function, acting as a co-gonadotropin and augmenting, in turn, ovarian function. Undoubtedly, the possible participation of the three previously suggested scenarios may also explain the amplified ovarian response shown by the glutamate-treated goats under the out-of-season reproductive response. These three scenarios generate new research questions while warranting new experimental challenges.

Glutamate is the main excitatory amino acid of the CNS, and its role as a neurotransmitter and neuromodulator is widely recognized in various brain processes [13]. Glutamate is synthesized and utilized as a substrate by various body tissues in key metabolic pathways for the proper functioning of the body [11,12]. Previous studies by our research team have demonstrated the role of exogenous glutamate and its positive effects on reproductive responses through the modulation of the synthesis of key metabolic and reproductive hormones [16,17,18,19]. Furthermore, the role of glutamate in the regulation of the function and survival of pancreatic endocrine cells has been unveiled in various animal models. Glutamate serves as a modulator of pancreatic function by interacting with ionotropic receptors located in the cell membrane, including N-methyl-D-aspartate (NMDA), α-amino-3-hydroxy-5-methyl-4-isoxazolepropionic acid (AMPA), and kainate receptor. These glutamate receptors are expressed in distinct pancreatic cell types (such as α, β, and γ cells), and upon activation, these receptors induce membrane depolarization by facilitating the opening of ion channels, leading to an influx of calcium ions into the intracellular environment [11,32]. Elevation in extracellular glutamate concentration activates the AMPA and kainate receptors; this activation evokes the stimulation of cGMP production, an increase in adenosine triphosphate (ATP) concentration, and the inhibition of ATP-sensitive K+ channels. Consequently, the plasma membrane is depolarized, and insulin secretion is activated in pancreatic β-cells [33]. 

In addition to participating in the regulation of glucose, insulin is also involved in the neuroendocrine regulation of the reproductive axis because insulin receptors are expressed along the reproductive axis, while the secretion of both GnRH and LH is stimulated in response to insulin administration [34,35]. Certainly, it has been proposed that insulin potentiates ovarian steroidogenesis in an LH-dependent fashion within ovarian theca cells [35,36]. The key role of insulin in reproductive function has been demonstrated in diabetic patients and in women with functional hypothalamic amenorrhea (FHA) who have hypogonadotropic hypogonadism and low fertility, respectively, with both pathologies coinciding with insufficient levels of insulin secretion [37,38,39]. 

The results obtained from our study indicate that the intravenous administration of exogenous glutamate resulted in an elevation of pancreatic glutamate concentration. This increase in glutamate concentration stimulated insulin secretion and may exert effects along with the HPG axis. Specifically, it is plausible that at the ovarian level, glutamate acts as a co-gonadotrophin, thereby enhancing the synthesis of ovarian sex steroids during the normal goat anestrous season at this latitude [36]. While our model supports the aforementioned response as the most compelling, it is important to acknowledge other intriguing pathways involving insulin and glutamate in the regulation of reproduction. Notably, insulin has been observed to exert its effect on non-neuronal populations by stimulating the release of gliotransmitters, specifically prostaglandin E2 (PGE2) [40,41,42,43]. This prostaglandin acts on GnRH neurons that express EP2 receptors, enhancing the secretion of GnRH [41,43]. Furthermore, it has been demonstrated that increased glutamate levels interact with astrocytes, leading to an elevation in PGE2 gliotransmission [15]. This mechanism potentially amplifies GnRH secretion, providing an additional link between glutamate and the modulation of reproductive processes.

This suggested mechanism of glutamate action on the pancreas to increase insulin release in conjunction with our results may be explained by the activation of a neuroendocrine pathway involving neuronal and glial cells, activating the reproductive axis. Additionally, exogenous glutamate has the potential to promote the reactivation of ovarian activity during seasonal anestrous, decreasing the inhibitory effect of estradiol negative feedback on the reproductive axis [16]. Under such a scenario, the augmented concentration of serum insulin observed in our study may have generated a positive influence on ovarian function [36]. 

**Figure 3:**
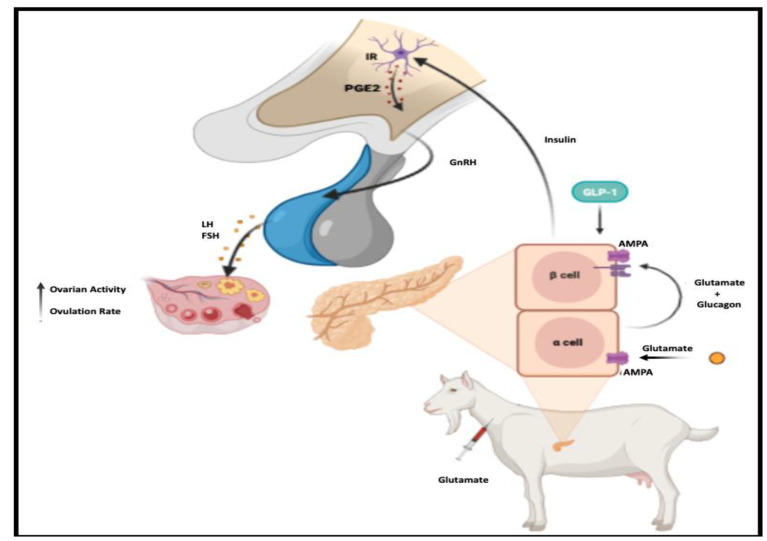
Proposed signaling pathway between insulin and hypothalamic astrocytes in response to exogenous glutamate signaling. The steps are explained in the text. Briefly, intravenous exogenous glutamate acts on glutamate receptors in pancreatic cells, increasing insulin secretion, which in turn enters the brain and binds to its receptor in hypothalamic astrocytes, generating an increase in GnRH and FSH and LH release, improving the ovarian activity in yearling goats during the anestrous season.

Certainly, it is necessary to conduct experiments to determine where exogenous glutamate acts within the reproductive axis. In addition, it is important to investigate whether the plausible effect mainly occurs at the brain level. This becomes particularly significant when considering that the majority of blood metabolites face significant challenges in crossing the blood–brain barrier (BBB) [44,45]. Further studies must, on the one hand, define whether and where glutamate reaches the brain and which neuronal populations might be activated, as well as what type of specific actions are triggered in response to exogenous glutamate. Another research approach could consider pharmacological studies using glutamate agonists and antagonists administered by different routes and therefore be able to better understand the dynamics of exogenous glutamate at different reproductive states. As for the suggested pathway involving the insulin’s action on hypothalamic astrocytes, it might be an alternative pathway that may help to explain the action of glutamate and its key role in modulating out-of-season reproductive neuroendocrinology and should therefore be investigated. Considering these findings and merging them with our research outcomes, a hypothetical signaling pathway can be suggested in which exogenous glutamate promotes insulin release, generating, in turn, an augmented ovarian activity through the possible release of gliotransmitters that modulate GnRH and LH secretion, a neuroendocrine scenario previously reported by our research group [19] (Figure 3).

Moreover, and from a biomedical and translational perspective, these results generate new opportunities to investigate reproductive failures related to metabolic disorders that coincide with insulin imbalances, as in the case of diabetic patients and women with FHA; additionally, hyperinsulinemia also generates reproductive problems, as in the case of women with polycystic ovary syndrome [32,35,36]. Therefore, understanding the mechanism of action of the glutamatergic system inside and outside the brain is central to the development of potential drugs to help the improvement of quality of life by decreasing the effects of metabolic imbalances and their deleterious actions upon reproductive function. All the proposed research approaches to solve the way and site of action of both exogenous glutamate administration linked to augmentations in serum insulin concentrations and an augmented ovarian function are, unquestionably, pending research assignments.

## 5. Conclusions

The obtained research outcomes support our working hypothesis; exogenous glutamate administration promoted an increased insulin concentration over the time, which was accompanied by augmentations in ovarian activity and ovulation rate in yearling dairy-type crossbred goats treated during the seasonal anestrous (i.e., April–June). Interestingly, the observed differences occurred between experimental groups irrespective of LW or BCS along with the experimental period. Understanding the mechanism of action of the glutamatergic insulin system either inside and/or outside the brain, considering a translational perspective, is central to the development of potential drugs that would help to improve quality of life through the lessening of neuro-metabolic imbalances and their interactions as well as their possible effects on ovarian function. Unquestionably, the use of inter-, multi-, and transdisciplinary approaches is certainly required to address such a crucial pending assignment.

## Figures and Tables

**Table 1:** Least squares means of live weight (LW), body condition score (BCS), ovulation rate (OR), and total ovarian activity (TOA) in glutamate-supplemented (GLUT) and non-supplemented (CONT) goat groups at the onset of treatments (INI) and ultrasound scanning (US).

	LW-INI (kg)	BCS-INI (Units)	LW-US (kg)	BCS-US (Units)	OR (Units)	TOA (Units)
GLUT	29.60 ^a^	3.40 ^a^	35.06 ^a^	3.50 ^a^	1.77 ^a^	4.11 ^a^
CONT	29.20 ^a^	3.50 ^a^	32.20 ^a^	3.20 ^a^	0.87 ^b^	1.87 ^b^
SE ^1^	1.02	1.07	0.17	0.20	0.20	0.47

^a,b^ Columns with distinct superscripts indicate significant differences (*p* < 0.05). ^1^ Most conservative standard error (SE) is presented.

## Data Availability

None of the data were deposited in an official repository, yet information can be made available upon request.
